# Aminotransferases in Relation to the Severity of Dengue: A Systematic Review

**DOI:** 10.7759/cureus.39436

**Published:** 2023-05-24

**Authors:** Pavan Kumar Reddy Kalluru, Mahesh Mamilla, Sai Sudha Valisekka, Saikiran Mandyam, Ernesto Calderon Martinez, Sarojini Posani, Shriya Sharma, Ravikishore Reddy Gopavaram, Borgharkar Gargi, Anvitha Gaddam, Sushritha Reddy

**Affiliations:** 1 Internal Medicine, Sri Venkateswara Medical College, Tirupati, IND; 2 Internal Medicine, University of Minnesota School of Medicine, Minneapolis, USA; 3 Internal Medicine, Southeast Health Medical Center, Dothan, USA; 4 Biomedical Informatics, Universidad Nacional Autónoma de México, Ciudad de Mexico, MEX; 5 Internal Medicine, Sri Devaraj Urs Medical College, Kothagudem, IND; 6 Internal Medicine, Dnipropetrovsk State Medical Academy, Dnipro, UKR; 7 Public Health, University of Alabama at Birmingham School of Medicine, Brimingham, USA; 8 Internal Medicine, Siddhartha Medical College, Vijayawada, IND; 9 Internal Medicine, Malla Reddy Institute of Medical Sciences, Hyderabad, IND

**Keywords:** dengue severity, dengue hemorrhagic fever (dhf), dengue fever/complications, aminotransferases, dengue

## Abstract

A systematic review was conducted to investigate the relationship between aminotransferases and the severity of dengue infection, which is a prevalent and significant infection in tropical and subtropical regions. Aminotransferases are enzymes that are often elevated in dengue due to the liver’s physiological and immunological response to the infection. This review focused on analyzing various studies that examined the correlation between aminotransferase levels and the severity of dengue. Extensive literature searches were performed using (“dengue*” OR “dengue fever*” OR “dengue haemorrhagic fever*” OR “dengue shock syndrome*”) AND (“alanine aminotransferase*” OR “aspartate aminotransferase*”) on PubMed. The selected articles were thoroughly reviewed, encompassing epidemiology, pathogenesis, and clinical manifestations of dengue. The consistent findings across the studies indicated that aminotransferases can serve as predictive markers for dengue severity. Therefore, early assessment of liver enzyme levels is crucial in dengue cases, and elevated levels should be closely monitored to prevent adverse outcomes.

## Introduction and background

Caused by four different dengue virus serotypes which are the members of the flavivirus family, dengue hemorrhagic fever is spread by the Aedes mosquito. Globally, and particularly in Southeast Asian nations, dengue fever is a severe public health hazard. Because of the wide range of organ involvement caused by dengue outbreaks, they are far more likely to cause morbidity and fatality [1]. Nearly 3,500 million people living in the tropics and subtropics are susceptible to this infection, with cases sometimes reaching up to 100 million reported yearly [2]. Most dengue infections are asymptomatic. The spectrum of symptomatic dengue fever includes fever without warning signs, fever with warning signs, and severe dengue. Traditional dengue fever develops in three stages, namely, the febrile phase, followed by the critical phase, and then the recovery phase. Although dengue is a self-limiting viral disease, a proportionate number of patients develop life-threatening complications as a result of this disease [3]. In dengue fever, hepatic impairment is frequently observed [4]. Aspartate aminotransferase (AST) and alanine aminotransferase (ALT) blood levels rise as a result of reactive hepatitis or the virus’s direct hepatocyte injury. Hepatic dysfunction shown as a rise in serum aminotransferase levels is associated with increased bleeding episodes, lower platelet count, shock, respiratory distress, and renal failure [4,5]. Therefore, the rise of serum aminotransferase levels is crucial in determining the severity of dengue fever. This study aims to determine the severity of dengue illness in relation to aminotransferases.

## Review

Methodology and results

Search Strategy

**Figure 1:**
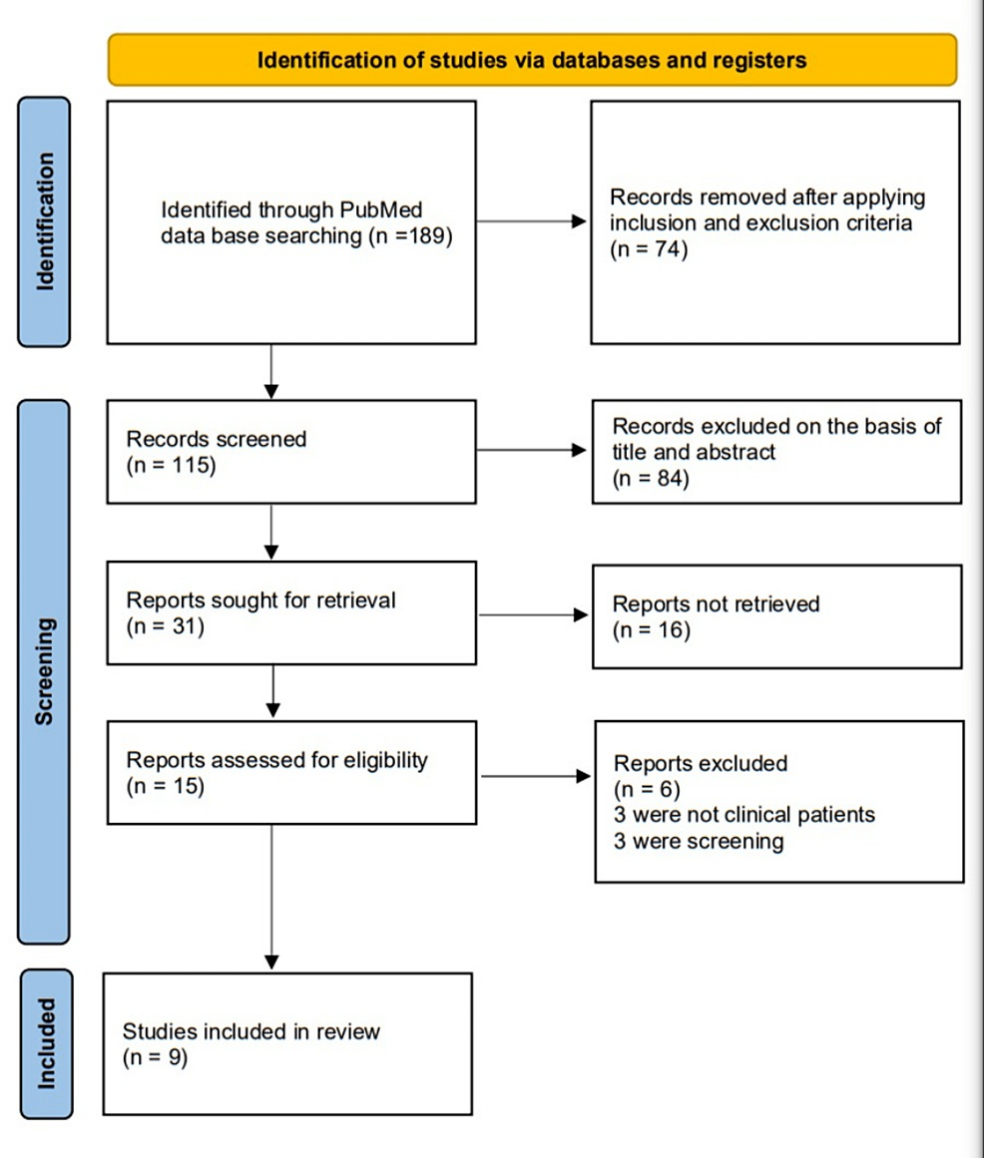
Preferred Reporting Items for Systematic Reviews and Meta-Analyses (PRISMA) 2009 flow diagram.

The search strategy and selection of studies for this systematic review are described in Figure 1 using the Preferred Reporting Items for Systematic Reviews and Meta-Analyses (PRISMA) 2009 flow diagram [6]. We conducted an extensive search through PubMed using the following: (“dengue*” OR “dengue fever*” OR “dengue haemorrhagic fever*” OR “dengue shock syndrome*”) AND (“alanine aminotransferase*” OR “aspartate aminotransferase*”).

Inclusion and Exclusion Criteria

The inclusion criteria for this review consist of studies published after the year 2000, specifically focusing on original research conducted on adult symptomatic human subjects. The studies should be written in English to ensure accessibility and comprehension. On the other hand, the exclusion criteria involve studies published on or before the year 2000 and studies conducted in languages other than English and on asymptomatic individuals. Additionally, letters to editors, literature reviews, and systematic and meta-analyses were excluded. Furthermore, studies involving animal subjects and those focused on children were also excluded.

Data Extraction and Analysis

The identified papers underwent a selection process based on predetermined inclusion and exclusion criteria. Titles and abstracts of potentially relevant articles were retrieved for further evaluation. After reading the full texts of the retrieved articles, those that met the inclusion criteria were chosen. To assess the risk of bias and the applicability of the selected studies, the Prediction Model Risk of Bias Assessment Tool (PROBAST) was employed [7]. The included publications were evaluated with regard to participant age, outcome measures, categorization of dengue severity, observation period, method of model construction, and variables considered. We gathered information on the study’s location, type, and year of publication, as well as the number of participants (Table 1). After a thorough analysis of the study, a column was set aside for comments on the study.

**Table 1:** Study findings. DF: dengue fever; DHF: dengue hemorrhagic fever; DSS: dengue shock syndrome; AST: aspartate aminotransferase; ALT: alanine aminotransferase

Authors	Country	Study	Year of publication	Sample size	Comment
Trung et al. [8]	Singapore	Prospective observational study	2010	644	AST and ALT levels were considerably more significant in individuals who had shock than those who did not during the crucial time, according to the link between transaminase levels and indicators of illness severity (P < 0.01 by the Mann-Whitney test)
Souza et al. [9]	Brazil	Retrospective observational study	2004	1585	These patients frequently had reactive hepatitis and liver damage as a result of the dengue virus infection and showed high AST and ALT
Parkash et al. [10]	Pakistan	Cohort comparative study	2010	699 patients categorized into DF/DHF/DSS	According to the study findings, severe hepatitis is a good predictor of dengue illness severity and is a bad prognostic indicator of outcome
Lee et al. [11]	Singapore	Retrospective observational study	2012	690 patients categorized into DF/DHF	Despite the fact that aminotransferase levels increased along with the severity of the dengue, increased aminotransferase values failed to distinguish DF from DHF or mild and severe dengue
Saha et al. [12]	India	Retrospective study	2013	1,226 patients categorized into DF/DHF	The research has shown that there are several cases of hepatic dysfunctions that resemble acute viral hepatitis
Rao et al. [13]	India	Prospective study	2020	106	The study demonstrated that serum AST and ALT levels and ferritin could serve as better biomarkers for determining the severity of the disease, and repeated estimation of these indicators should be taken into consideration
Md Sani et al. [14]	Malaysia	Retrospective cohort study	2017	365 patients	The composite index, AST^2^/ALT can be employed as a marker for recognizing severe dengue, with two options for cut-off values: 402 and 653
Huy al. [15]	Vietnam	Multicentric cross-sectional study	2022	326 patients categorized as DF/DHF	Platelet count or serum albumin can be used as prognostic indications during the first three days of sickness. Prognostic indications should be AST, ALT, albumin, or total bilirubin from the fourth to the sixth day of clinical symptoms
Srisuphanunt et al. [16]	Thailand	Retrospective study	2022	302	The study suggested that patients with dengue infection could have their disease severity predicted using aminotransferases in combination with other parameters

Discussion

Epidemiology and History

Dengue virus, an arbovirus, belongs to the Flaviviridae family and flavivirus genus. Four different dengue virus serotypes (DENV 1-4) have been identified and are transmitted by the *Aedes *mosquito [17,18]. It is the most prevalent arboviral disease globally, with tropical and subtropical areas bearing the brunt of its impact [19]. During the Caribbean exanthema epidemic accompanying arthralgia in 1827-1828, the name Dengue was first used in English-language medical literature, the Spanish equivalent of the Swahili term *Ki denga Pepo*, which means an unexpected cramp-like spasm brought on by an evil spirit. Since 1780, Dengue has been referred to as the *Break bone fever*.

Around 33% of the earth’s population is at risk of dengue infection [19]. Tropics and subtropics are at an increased risk compared to other regions. In some nations, the prevalence of infection follows a predictable pattern, low temperatures reduce the survival of adult mosquitoes, thus impacting transmission rates. Rain and temperature change the mosquito’s reproduction and feeding rhythm, which impacts the density of the vector population [20]. Dengue fever, which is spread by mosquitoes, was the second most important tropical infectious disease in 1998, after malaria, with an estimated 100 million illnesses, 500,000 cases of dengue hemorrhagic fever, and 25,000 fatalities annually. In the past 20 years, dengue has expanded to new geographical locations. Reasons for this expansion to new geographic areas are mainly attributed to changes in public health infrastructure and demographics over the past 30 years [21].

Pathophysiology

The four distinct dengue virus serotypes comprise a phylogenetic group and vary from one another in terms of nucleotide sequence [22]. The dengue virus genome consists of seven non-structural (NS) proteins, including NS1, NS2A, NS2B, NS3, NS4A, NS4B, and NS5, as well as three structural protein genes that encode nucleocapsid of the core protein (C), a membrane-associated protein (M), and an envelope protein (E). The host immune system and the NS1 protein interact, which causes a T-cell response. Individuals with this infection have detectable amounts of the NS1 protein in their serum, which is a diagnostic indicator [23]. Although the precise pathogenesis of dengue fever is unclear, the clinical events can be distinguished by early infection, propagation of the virus, immunological response, and subsequent viral clearance [24]. Additionally, essential to the pathophysiology of dengue fever is the host immunological response. Most of the explanations for the signs and symptoms explained above center around one of the following three concepts: antibody-dependent enhancement (ADE), vasculopathy, cytokine storm, and coagulopathy [25].

Dendritic cells initially engulf the dengue virus, and after digesting it, they present the T cells with an antigen. The host immune system explicitly targets the E protein, precursor membrane protein (Pre-M), and NS1 as the three main proteins of the dengue virus. E protein-specific antibodies prevent binding to cell receptors and neutralize the infection. NS1-specific antibodies can drive complement-mediated lysis of infected cells but are ineffective in neutralizing the infection. Pre-M-specific antibodies can promote ADE but only exhibit partial binding to matured virions and have insufficient neutralization [26,27].

Neutralizing and non-neutralizing antibodies are created due to the invasion of the dengue virus. The neutralizing antibodies can offer a defense against a particular viral serotype. Non-neutralizing antibodies bind to an infecting virus; however, they cannot neutralize it. Instead, they facilitate the pathogen’s entry into the cell [28]. Within the cell, viral replication begins and raises blood virus titers. ADE of infection is the term used to describe this phenomenon. Dengue-specific CD4+ and CD8+ T cells release interferon-gamma (IFN-gamma), tumor necrosis factor-alpha (TNF-alpha), and lymphotoxin, which cause a cytokine storm and, eventually, further deterioration of the illness. Immunoglobulin receptor expression is also increased by IFN-gamma, which increases the antibody-dependent intensification of the infection [28].

Vasculopathy and coagulopathy are the two pathways of pathogenesis that support the clinical manifestation of dengue. Most symptomatic patients recover after a brief illness; a small percentage of patients have more severe sickness, which often presents as a vasculopathy with hemorrhagic diathesis and plasma leakage. The infection causes a small modification in the fiber matrix properties of the endothelium by temporarily disrupting the function of the endothelial glycocalyx layer. As autoantibodies, anti-NS1 antibodies interact with platelets and uninfected endothelial cells to cause intracellular signaling that alters capillary permeability. Plasma leakage leading to ascites, hemoconcentration, and effusions in pleural and pericardial spaces are brought on by a diffuse increase in capillary permeability. The severity of vasculopathy is correlated with the degree of organ involvement and shock. Typically, it shows up between the third and seventh day of the disease [25,26]. Raised activated partial thromboplastin time and low fibrinogen levels are generally regular observations. Thrombocytopenia as an outcome of coagulation defect is associated with worse features. Coagulation disorder is also exacerbated by releasing heparan sulfate or chondroitin sulfate from the glycocalyx, two molecules with structural similarities to heparin that can perform its anticoagulant action [27].

The immune enhancement and virus virulence hypothesis has been proposed to explain why severe disease occurs. Dengue shock syndrome is more frequent in people exposed to secondary dengue infection caused by a different dengue virus serotype. ADE therapy can explain this phenomenon. Complexes of prior non-neutralizing antibodies develop inside the viral particles. During secondary dengue virus infections, they boost their absorption and replication in the body’s macrophage system. T cells, mast cells, and monocytes create more cytokines when infected with dengue. INF-gamma, TNF-alpha, interleukin (IL)-2, and IL-6 are at their peak levels during the first three days of sickness, while an increase in IL-4, IL-5, and IL-10 is seen after three days [29].

Clinical Manifestations

Dengue with or without warning signs and severe dengue are included in the 2009 WHO classification changes. Dengue without warning signs includes fever, two skin rashes, bodily aches, nausea, vomiting, or retro-orbital pain. Dengue fever with warning signs includes fever with any two of the skin rash, bodily pains, nausea, or vomiting, and any one of the warning signs, which include abdominal tenderness, frequent vomiting, signs of fluid retention, such as effusions and ascites, mucosal bleeding, restlessness, lethargy, rise in hematocrit (20%) with a sharp decline in thrombocyte count (50,000/mm^3^), and liver enlargement >2 cm. The following three features characterize severe dengue: (1) Severe plasma leakage that results in oliguria, shock, or a delay in capillary filling and/or fluid build-up in the serosa leading to breathing difficulties. (2) Extreme bleeding symptoms. (3) Significant organ involvement: more than 1,000 units of AST or ALT, hepatomegaly and liver failure, consciousness impairment, and myocardial malfunction [30,31].

The symptoms of dengue fever, an acute febrile sickness, include muscle, bone, and joint pains; headaches; leukopenia; and rash. Severe fever, hemorrhage, frequently accompanied by hepatomegaly, and, in severe cases, circulatory failure are the main clinical symptoms of dengue hemorrhagic fever. Dengue hemorrhagic fever can occasionally lead to the potentially fatal dengue shock syndrome, which has features similar to anaphylaxis [32,33].

Dengue fever follows a course of illness consisting of three phases, namely, the febrile phase, the critical phase, and the recovery phase. During the febrile phase, which lasts around three to five days, the symptoms resemble those of other febrile diseases, such as body aches, headaches, joint pain, and loss of appetite. A notable indicator during this phase is a gradual reduction in the white blood cell count, which raises concerns about dengue. The critical phase is characterized by an abnormal increase in capillary permeability, leading to plasma leakage and a rise in hematocrit levels. This phase lasts from 24 to 48 hours and is accompanied by progressive leukopenia and a rapid decline in platelet counts. Warning signs often precede shock, and impairment of the organ systems, metabolic acidosis, and disseminated intravascular coagulation can be seen. The recovery phase begins after the critical phase, during which extravascular fluid is gradually absorbed. The patient’s general condition and hemodynamic status improve, and white blood cell counts return to normal once platelet counts have normalized. Avoiding excessive fluid management during this phase is essential to prevent complications such as pulmonary edema or congestive cardiac failure [34,35].

Prevention and Control

No specific medication or vaccination is available today to protect against the dengue virus. Therefore, control of the vector is a critical factor in controlling infection, including environmental, biological, and chemical control, apart from personal protective measures. Subjective measures include protective clothing, insect repellents, mosquito nets, and curtains treated with pesticides. Environmental measures include adequate provision of drinking water, proper roofing of overhead tanks, and an underground drainage system. Gambia and Peorilia reticulate, long-lived fish usage come under biological control. Chemical control uses malathion-based space sprayers and 1% temephos granules. Additionally, insect growth regulators can be used [36].

Lately, dengue vaccinations have gained much focus, with research in this area yielding promising results. However, the difficulty of performing long-term trials to evaluate vaccination efficacy and safety to eliminate the danger of vaccine-induced dengue hemorrhagic fever/dengue shock syndrome, particularly in children, has complicated efforts to create safe and effective vaccines to prevent dengue infections. At least seven vaccines have been tested in various stages of clinical trials, but just three (Dengvaxia, TV003, and TAK-003) have shown promising outcomes [37].

Dengue Fever and Aminotransferases

Hepatic injury is common in dengue infection, ranging from mild elevations in hepatic transaminases to severe acute liver failure (ALF) with fatal outcomes. While viral hepatitis and drugs are the leading causes of ALF, infectious diseases such as dengue are also recognized as etiological factors [38]. Various manifestations characterize liver injury in dengue. Hepatocytes and Kupffer cells are the primary targets of the dengue virus. The initial step in the viral infection process involves viral attachment to receptors on the host cell surface, with the E protein playing a crucial role in this attachment. It has been observed that the binding of dengue viruses to hepatocytes facilitates the subsequent binding of viral particles. Following viral attachment, the internalization of the virus occurs through direct fusion or endocytosis [38].

The pathogenesis of hepatic injury in dengue is primarily believed to be T-cell-mediated. The interaction between antibodies, the endothelium, and the concurrent cytokine storm is crucial. CD4+ cytotoxic T cells have been identified as responsible for liver dysfunction in dengue fever, involving mechanisms such as bystander lysis [39]. The pathogenesis of liver injury in dengue involves the virus’s direct effect on hepatocytes and immune-mediated hepatitis. During acute dengue attack, a varying range of liver involvement can be observed, such as apoptosis of liver cells induced by the dengue virus, hypoxic damage from altered perfusion, oxidative stress, or immunological injury. Elevated serum aminotransferase levels are commonly seen in hepatic involvement in dengue fever. Such patients are at a higher risk of bleeding. Acute respiratory distress syndrome, cholecystitis, shock, renal failure, and hepatic dysfunction play a significant role in bleeding [39]. Gagnon et al. [40] demonstrated that CD4+ T cells contribute to the destruction of hepatocytes in dengue fever. This is mediated by two pathways, namely, perforin and granzymes released by activated CD4+ T cells, and the interaction between Fas and Fas ligands on target and T cells, respectively [41,42].

The findings from various studies indicate that elevated liver enzymes, specifically AST and ALT, are commonly observed in patients with dengue fever. These studies have demonstrated significant liver dysfunction in over 50% of individuals diagnosed with dengue fever [43,44]. In addition, liver dysfunction rates are high in Asian populations ranging from 30% to 90% [45]. These studies also observed that increased AST and ALT were associated with severe bleeding manifestations. Additionally, it should be noted that vomiting from the first day of illness could indicate possible hepatic dysfunction. Early-onset vomiting may be a clinical sign that warrants attention to the liver function of dengue fever patients [8]. The mean values of AST and ALT have been observed to be higher in severe dengue than in dengue fever without warning signs, suggesting a correlation between increased transaminase levels and disease severity. AST is derived from various sources, including the liver, heart, striated muscle, and erythrocytes, whereas ALT is primarily hepatic. Non-hepatic tissue damage caused by the dengue virus can contribute to higher AST levels than ALT, indicating that AST elevations may not solely reflect liver involvement [46,47].

A negative correlation between liver enzyme levels and platelet count indicated a potential relationship between liver dysfunction and thrombocytopenia. In addition, several studies significantly associated bleeding tendencies with elevated liver enzymes [11,48]. Hepatomegaly was found in some patients, and elevated AST levels were detected in all cases of hepatomegaly. However, there was no significant association between elevated liver enzymes and free fluid in the abdomen. Ultrasound findings in dengue fever often include liver enlargement, thickening of the gallbladder, and third-space fluid build-up [49]. Moreover, it should be noted that there were no significant gender or age differences in terms of transaminase elevation levels in the studies [10,50]. In addition, jaundice, a known hepatic alteration in dengue infection, was reported in only a minority of patients [51].

## Conclusions

Hepatic dysfunction in dengue fever can result from direct viral damage to the liver cells or reactive hepatitis. The elevation of liver enzymes typically occurs between the third and fifth day of fever onset. Patients with elevated liver enzymes during the febrile phase are at a higher risk of developing complications and require careful management during the critical phase. The study’s findings indicate a strong association between increased levels of liver enzymes, specifically AST and ALT, and the severity of dengue fever. Furthermore, the research suggests that patients with elevated liver enzymes are more likely to exhibit bleeding tendencies, hepatomegaly (enlarged liver), and the presence of fluid in the abdomen, providing additional support for the conclusion. Therefore, early evaluation of liver enzyme levels is crucial in all cases of dengue fever, and patients with elevated levels should receive close monitoring to prevent unfavorable outcomes.
